# Logical Entropy of Information Sources

**DOI:** 10.3390/e24091174

**Published:** 2022-08-23

**Authors:** Peng Xu, Yamin Sayyari, Saad Ihsan Butt

**Affiliations:** 1 School of Computer Science of Information Technology, Qiannan Normal University for Nationalities, Duyun 558000, China; 2 Department of Mathematics, Sirjan University of Technology, Sirjan 7813733385, Iran or; 3 Department of Mathematics, COMSATS University Islamabad, Lahore Campus, Islamabad 54000, Pakistan

**Keywords:** entropy, logical entropy, random variable, information source, convex function, 94A17, 37B40, 26A51, 81P10

## Abstract

In this paper, we present the concept of the logical entropy of order *m*, logical mutual information, and the logical entropy for information sources. We found upper and lower bounds for the logical entropy of a random variable by using convex functions. We show that the logical entropy of the joint distributions $X_{1}$ and $X_{2}$ is always less than the sum of the logical entropy of the variables $X_{1}$ and $X_{2}$. We define the logical Shannon entropy and logical metric permutation entropy to an information system and examine the properties of this kind of entropy. Finally, we examine the amount of the logical metric entropy and permutation logical entropy for maps.

## 1. **Introduction and Basic Notions**

Entropy is an influential quantity that has been explored in a wide range of studies, from applied to physical sciences. In the 19th century, Carnot and Clausius diversified the concept of entropy into three main directions—entropy associated with heat engines (where it behaves similar to a thermal charge), statistical entropy, and (according to Boltzmann and Shannon) entropy in communications channels and information security. Thus, the theory of entropy plays a key role in mathematics, statistics, dynamical systems (where complexity is mostly measured by entropy), information theory [1], chemistry [2], and physics [3] (see also [4,5,6]).

In recent years, other information source entropies have been studied [7,8,9]. Butt et al. in [10,11] introduced new bounds for Shannon, relative, and Mandelbrot entropies via interpolating polynomials. Amig and colleagues defined entropy as a random process and the permutation entropy of a source [1,12].

Ellerman [13] was the first to take credit for introducing a detailed introduction to the concept of logical entropy and establishing its relationship with the renowned Shannon entropy. In recent years, many researchers have focused on extending the notion of logical entropy in new directions/perspectives. Markechová et al. [14] proposed the study of logical entropy and logical mutual information of experiments in the intuitionistic fuzzy case. Ebrahimzadeh [15] proposed the logical entropy of a quantum dynamical system and investigated its ergodic properties. However, the logical entropy of a fuzzy dynamical system was investigated in [7] (see also [16]). Tamir et al. [17] extended the idea of logical entropy over the quantum domain and expressed it in terms of the density matrix. In [18], Ellerman defined logical conditional entropy and logical relative entropy. In fact, logical entropy is a particular case of Tsallis entropy when q=2. Logical entropy resembles the information measure introduced by Brukner and Zeilinger [19]. In [13], Ellerman introduced the concept of logical entropy for a random variable. He studied the logical entropy of the joint distribution p(x,y) over X×Y as:

$$\begin{array}{c} h\left(x,y\right)=1-\sum_{x,y}{\left[p\left(x,y\right)\right]}^{2}. \end{array}$$

The motive of this study was to extend the concept of logical entropy presented in [13] to information sources. Since estimating entropy from the information source can be difficult [20], we defined the logical metric permutation entropy of a map and used it to apply for an information source.

In the article, (Γ,G,μ) is a measurable probability space (i.e., Γ≠∅ and G enjoys the structure of σ-algebra of subsets of Γ with μ(Γ)=1). Further, if *X* is a random variable of Γ possessing discrete finite state space $A=\{a_{1},\ldots ,a_{n}\}$, then the function p:A→[0,1] defined by

$$\begin{array}{c} p(x)=\mu \{\gamma \in \Gamma :X(\gamma )=x\} \end{array}$$

is a probability function. $H_{\mu }\left(X\right)=-\sum_{x\in A}p\left(x\right)\log p\left(x\right)$ denotes the Shannon entropy of *X* [1]. If ${\left(X_{n}\right)}_{n=1}^{\infty }$ is a sequence of the random variables on Γ, the sequence $X_{n}$ is called an information source (also called the stochastic process [S.P]). Similarly, if m≥1, then we define $p:A^{m}\to \left[0,1\right]$ by

$$\begin{array}{c} p\left(x_{1},\ldots ,x_{m}\right)=\mu \left\{\gamma \in \Gamma :X_{1}\left(\gamma \right)=x_{1},\ldots ,X_{m}\left(\gamma \right)=x_{m}\right\}. \end{array}$$

We know that

$$\begin{array}{c} \sum_{x_{1},\ldots ,x_{m}\in A}p\left(x_{1},\ldots ,x_{m}\right)=\mu \left(\Gamma \right)=1 \end{array}$$

for every natural number *m*. A finite space S.P, $X={\left(X_{n}\right)}_{n=1}^{\infty }$ can be recalled as a stationary finite space S.P if

$$\begin{array}{c} p\left(x_{1},\ldots ,x_{m}\right)=\mu \left\{\gamma \in \Gamma :X_{k+1}\left(\gamma \right)=x_{1},\ldots ,X_{k+m}\left(\gamma \right)=x_{m}\right\}, \end{array}$$

for every m,k∈N. In an information–theoretical setting, one may assume a stationary S.P, X as a data source. A finite space S.P, X is strictly a stationary finite space S.P if

$$\begin{array}{c} p\left(x_{1},\ldots ,x_{m}\right)=\mu \left\{\gamma \in \Gamma :X_{k_{1}}\left(\gamma \right)=x_{1},\ldots ,X_{k_{m}}\left(\gamma \right)=x_{m}\right\}, \end{array}$$

for every $\{k_{1},\ldots ,k_{m}\}\subseteq N$. The Shannon entropy of order *m* of source X is defined by [1,12]

$$\begin{array}{c} H_{\mu }\left(X_{1}^{m}\right)=-\sum_{x_{1},\ldots ,x_{m}\in A}p\left(x_{1},\ldots ,x_{m}\right)\log p(x_{1},\ldots ,x_{m}). \end{array}$$

The Shannon entropy of source X is defined by $h_{\mu }\left(X\right)=\lim_{m\to \infty }\left(\frac{1}{m}H_{\mu }\left(X_{1}^{m}\right)\right).$ If we assume that the alphabet *A* from source X accepts an order ≤, so that (A,≤) is a totally ordered set, then define another order ≺ on *A* by [1]

$$\begin{array}{c} t_{i}≺t_{j}\Leftrightarrow t_{i}<t_{j}\;\mathrm{or}\;\left(t_{i}=t_{j}\;\mathrm{and}\;i<j\right). \end{array}$$

We say that a length-*m* sequence $t_{k}^{k+m-1}=\left(t_{k},\ldots ,t_{k+m-1}\right)$ has an order pattern π if, $t_{k+\pi (0)}≺t_{k+\pi (1)}≺\ldots ≺t_{k+\pi (m-1)},$ where $t_{i},t_{j}\in A$, k∈N and i≠j. To a S.P, $X={\left(X_{n}\right)}_{n\in N_{0}}$ we associate a probability process $R={\left(R_{n}\right)}_{n\in N_{0}}$ defined by $R_{m}\left(\gamma \right)=\sum_{i=0}^{m}\delta \left(X_{i}\left(\gamma \right)\leq X_{m}\left(\gamma \right)\right).$ The sequence R defines a discrete-time process that is non-stationary. The metric permutation entropy of order *m* and the metric permutation entropy of source X are, respectively, defined by [1,12]

$$\begin{array}{c} H_{\mu }^{★}\left(X_{0}^{m-1}\right)=H_{\mu }\left(R_{0}^{m-1}\right)=\frac{-1}{m-1}\sum_{r_{0},\ldots ,r_{m-1}}p\left(r_{0}^{m-1}\right)\log p\left(r_{0}^{m-1}\right), \end{array}$$

and $h_{\mu }^{★}\left(X\right)={lim sup}_{m\to \infty }H_{\mu }^{★}\left(X_{0}^{m-1}\right).$

## 2. **Main Results**

In this section, we use the symbol $x_{1}^{m}$ for $\left(x_{1},\ldots ,x_{m}\right)$ to simplify the notation.

**Definition** **1.** *Reference [13]. Let X be a random variable on *Γ* with discrete finite state space $A=\{a_{1},\ldots ,a_{n}\}$. Then,*

$$\begin{array}{c} H_{\mu l}\left(X\right)=\sum_{x\in A}p\left(x\right)\left[1-p\left(x\right)\right]=1-\sum_{x\in A}{\left[p\left(x\right)\right]}^{2} \end{array}$$

*is called the logical Shannon entropy of X.*

**Theorem** **1.** *Reference [21] If f is convex on I and $\zeta =\min_{1\leq i\leq n}\left\{y_{i}\right\}$, $\eta =\max_{1\leq i\leq n}\left\{y_{i}\right\}$, then*

$$\begin{array}{c} \frac{f\left(\zeta \right)+f\left(\eta \right)-2f\left(\frac{\zeta +\eta }{2}\right)}{n}\leq \frac{\sum_{i=1}^{n}f\left(y_{i}\right)}{n}-f\left(\frac{\sum_{i=1}^{n}y_{i}}{n}\right)\leq f\left(\zeta \right)+f\left(\eta \right)-2f\left(\frac{\zeta +\eta }{2}\right). \end{array}$$

**Theorem** **2.** *Suppose that X is a random variable on *Γ* with a discrete finite state space $A=\{a_{1},\ldots ,a_{n}\}$, $\zeta =\min_{1\leq i\leq n}\left\{p\left(a_{i}\right)\right\}$ and $\eta =\max_{1\leq i\leq n}\left\{p\left(a_{i}\right)\right\}$, then*

$$\begin{array}{c} 0\leq \Delta \left(\zeta ,\eta \right):=\frac{{\left(\zeta -\eta \right)}^{2}}{4}\leq \frac{n-1}{n}-H_{\mu l}\left(X\right)\leq n\frac{{\left(\zeta -\eta \right)}^{2}}{4}=n\Delta \left(\zeta ,\eta \right). \end{array}$$

**Proof.**  Applying Theorem 1 with $f\left(x\right)=x^{2}-x$, we obtain

$$\begin{array}{cc} \; & \frac{1}{n}\left(\left(\zeta^{2}-\zeta \right)+\left(\eta^{2}-\eta \right)-2\left({\left(\frac{\zeta +\eta }{2}\right)}^{2}-\frac{\zeta +\eta }{2}\right)\right)\\ \; & \leq \frac{1}{n}\sum_{i=1}^{n}\left(x_{i}^{2}-x_{i}\right)-\left({\left(\frac{\sum_{1}^{n}x_{i}}{n}\right)}^{2}-\left(\frac{\sum_{1}^{n}x_{i}}{n}\right)\right)\\ \; & \leq \left(\zeta^{2}-\zeta \right)+\left(\eta^{2}-\eta \right)-2\left({\left(\frac{\zeta +\eta }{2}\right)}^{2}-\frac{\zeta +\eta }{2}\right). \end{array}$$

Putting $y_{i}=p\left(a_{i}\right)$, it follows that

$$\begin{array}{cc} \; & \frac{1}{n}\left(\left(\zeta^{2}-\zeta \right)+\left(\eta^{2}-\eta \right)-2\left({\left(\frac{\zeta +\eta }{2}\right)}^{2}-\frac{\zeta +\eta }{2}\right)\right)\\ \; & \leq \frac{1}{n}\sum_{i=1}^{n}\left({\left(p\left(a_{i}\right)\right)}^{2}-p\left(a_{i}\right)\right)-\left({\left(\frac{\sum_{1}^{n}p\left(a_{i}\right)}{n}\right)}^{2}-\left(\frac{\sum_{1}^{n}p\left(a_{i}\right)}{n}\right)\right)\\ \; & \leq \left(\zeta^{2}-\zeta \right)+\left(\eta^{2}-\eta \right)-2\left({\left(\frac{\zeta +\eta }{2}\right)}^{2}-\frac{\zeta +\eta }{2}\right). \end{array}$$

Thus,

$$\begin{array}{cc} \; & \frac{1}{n}\left(\left(\zeta^{2}-\zeta \right)+\left(\eta^{2}-\eta \right)-2\left({\left(\frac{\zeta +\eta }{2}\right)}^{2}-\frac{\zeta +\eta }{2}\right)\right)\\ \; & \leq \frac{1}{n}\sum_{i=1}^{n}{\left(p\left(a_{i}\right)\right)}^{2}-\frac{1}{n}\sum_{i=1}^{n}p\left(a_{i}\right)-\left(\frac{1}{n^{2}}-\frac{1}{n}\right)\\ \; & \leq \left(\zeta^{2}-\zeta \right)+\left(\eta^{2}-\eta \right)-2\left({\left(\frac{\zeta +\eta }{2}\right)}^{2}-\frac{\zeta +\eta }{2}\right). \end{array}$$

Hence,

$$\begin{array}{cc} \; & \frac{1}{n}\left(\left(\zeta^{2}-\zeta \right)+\left(\eta^{2}-\eta \right)-2\left({\left(\frac{\zeta +\eta }{2}\right)}^{2}-\frac{\zeta +\eta }{2}\right)\right)\\ \; & \leq \frac{1}{n}\left(1-H_{\zeta l}\left(X\right)\right)-\frac{1}{n}-\left(\frac{1}{n^{2}}-\frac{1}{n}\right)\\ \; & \leq \left(\zeta^{2}-\zeta \right)+\left(\eta^{2}-\eta \right)-2\left({\left(\frac{\zeta +\eta }{2}\right)}^{2}-\frac{\zeta +\eta }{2}\right). \end{array}$$

After some calculations, it turns out that

$$\begin{array}{c} \Delta \left(\zeta ,\eta \right):=\frac{{\left(\zeta -\eta \right)}^{2}}{4}\leq \frac{n-1}{n}-H_{\mu l}\left(X\right)\leq n\frac{{\left(\zeta -\eta \right)}^{2}}{4}. \end{array}$$

□

**Lemma** **1.** *Let X be a random variable with alphabet $A=\{a_{1},\ldots ,a_{n}\}$. Then, $0\leq H_{\mu l}\left(X\right)\leq \frac{n-1}{n}$, and equality holds if and only if $p\left(a_{i}\right)=p\left(a_{j}\right)$ for every 1≤i,j≤n.*

**Proof.**  Using Theorem 2, we obtain $0\leq H_{\mu l}\left(X\right)\leq \frac{n-1}{n}$. Now, let $H_{\mu l}\left(X\right)=\frac{n-1}{n}$, by the use of Theorem 2, we have $M\left(\zeta ,\eta \right)=\frac{{\left(\zeta -\eta \right)}^{2}}{4}=0$ and, thus, ζ=η. Therefore, $\max_{1\leq i\leq n}\left\{p\left(a_{i}\right)\right\}=\min_{1\leq i\leq n}\left\{p\left(a_{i}\right)\right\}$. Thus, $p\left(a_{i}\right)=p\left(a_{j}\right)$ for every 1≤i,j≤n. On the other hand, if $p\left(a_{i}\right)=p\left(a_{j}\right)$ for every 1≤i,j≤n, then ζ=η, so M(ζ,η)=0 and by the use of Theorem 2, we obtain $H_{\mu l}\left(X\right)-\frac{n-1}{n}=0$. Hence, $H_{\mu l}\left(X\right)=\frac{n-1}{n}$. □

**Definition** **2.** *The logical Shannon entropy of order m of source X is defined by*

$$\begin{array}{cc} H_{\mu l}\left(X_{1}^{m}\right)=H_{\mu l}\left(X_{1},\ldots ,X_{m}\right) & :=\sum_{x_{1},\ldots ,x_{m}\in A}p\left(x_{1},\ldots ,x_{m}\right)(1-p(x_{1},\ldots ,x_{m})),\\ & =1-\sum_{x_{1},\ldots ,x_{m}\in A}(p\left(x_{1},\ldots ,x_{m}\right){)}^{2} \end{array}$$

It is easy to see that may be $p\left(x_{1},x_{2}\right)\neq p\left(x_{2},x_{1}\right)$ but for every two random variables $x_{1},x_{2}$ we have $H_{\mu l}\left(x_{1},x_{2}\right)=H_{\mu l}\left(x_{2},x_{1}\right)$.

**Definition** **3.** *Let m be a natural number and $1\leq i_{1},\ldots ,i_{m}\leq n$. We define the sets $A_{i_{1}i_{2}\ldots i_{m}}$ by*

$$\begin{array}{c} A_{i_{1}i_{2}\ldots i_{m}}=\left\{\gamma \in \Gamma :X_{1}\left(\gamma \right)=a_{i_{1}},X_{2}\left(\gamma \right)=a_{i_{2}},\ldots ,X_{m}\left(\gamma \right)=a_{i_{m}}\right\}. \end{array}$$

*and $\mu \left(A_{i_{1}i_{2}\ldots i_{m}}\right):=a_{i_{1}i_{2}\ldots i_{m}}$.*

Moreover, $A_{i_{1}i_{2}\ldots i_{m}}\cap A_{j_{1}j_{2}\ldots j_{m}}=\emptyset$ for every $\left(i_{1},i_{2},\ldots ,i_{m}\right)\neq \left(j_{1},j_{2},\ldots ,j_{m}\right)$ and for every m∈N. Furthermore, if $\gamma \in {⋃}_{j=1}^{n}A_{i_{1}i_{2}\ldots i_{m}j}$, then $\gamma \in A_{i_{1}i_{2}\ldots i_{m}j_{0}}$ for some $j_{0}\in \left\{1,\ldots ,n\right\}$. Hence,

$$\begin{array}{c} X_{1}\left(\gamma \right)=a_{i_{1}},\ldots ,X_{m}\left(\gamma \right)=a_{i_{m}},X_{m+1}\left(\gamma \right)=a_{j_{0}} \end{array}$$

for some $j_{0}\in \left\{1,\ldots ,n\right\}$ and, thus, $\gamma \in A_{i_{1}i_{2}\ldots i_{m}}$. Moreover, if $\gamma \in A_{i_{1}i_{2}\ldots i_{m}}$, then

$$\begin{array}{c} X_{1}\left(\gamma \right)=a_{i_{1}},\ldots ,X_{m}\left(\gamma \right)=a_{i_{m}}. \end{array}$$

Define $X_{m+1}\left(\gamma \right)=a_{j_{0}}$. Therefore,

$$\begin{array}{c} X_{1}\left(\gamma \right)=a_{i_{1}},\ldots ,X_{m}\left(\gamma \right)=a_{i_{m}},X_{m+1}\left(\gamma \right)=a_{j_{0}}. \end{array}$$

Hence, $\gamma \in A_{i_{1}i_{2}\ldots i_{m}j_{0}}$ for some $j_{0}\in \left\{1,\ldots ,n\right\}$ and, thus, $\gamma \in {⋃}_{j=1}^{n}A_{i_{1}i_{2}\ldots i_{m}j}$. So,

$$\begin{array}{c} A_{i_{1}i_{2}\ldots i_{m}}=\overset{n}{\underset{j=1}{⋃}}A_{i_{1}i_{2}\ldots i_{m}j} \end{array}$$

and, therefore, $\Gamma ={⋃}_{i_{1},i_{2},\ldots ,i_{m}}A_{i_{1}i_{2}\ldots i_{m}}$. Hence, we obtain

$$\begin{array}{c} \sum_{i_{1}i_{2}\ldots i_{m}}a_{i_{1}i_{2}\ldots i_{m}}=1 \end{array}$$

and

(1) $$\begin{array}{cc} a_{i_{1}i_{2}\ldots i_{m}} & =\mu \left(A_{i_{1}i_{2}\ldots i_{m}}\right)=\mu \left(\cup_{j=1}^{n}A_{i_{1}i_{2}\ldots i_{m}j}\right)\\ \; & =\sum_{j=1}^{n}\mu \left(A_{i_{1}i_{2}\ldots i_{m}j}\right)=\sum_{j=1}^{n}a_{i_{1}i_{2}\ldots i_{m}j} \end{array}$$

for every $1\leq i_{1},i_{2},\ldots ,i_{m}\leq n$.

We now prove the following Theorem by employing Lemma A1 (see Appendix A):

**Theorem** **3.** *If $X_{1}$ and $X_{2}$ are two random variables on *Γ*, then*

(2) $$\begin{array}{c} \max\left\{H_{\mu l}\left(X_{1}\right),H_{\mu l}\left(X_{2}\right)\right\}\leq H_{\mu l}\left(X_{1},X_{2}\right)\leq H_{\mu l}\left(X_{1}\right)+H_{\mu l}\left(X_{2}\right). \end{array}$$

**Proof.**  Suppose $A=\{a_{1},\ldots ,a_{n}\}$. For every 1≤i,j≤n, we consider

$$\begin{array}{cc} \; & B_{i}=\left\{\gamma \in \Gamma :X_{1}\left(\gamma \right)=a_{i}\right\},\;C_{j}=\left\{\gamma \in \Gamma :X_{2}\left(\gamma \right)=a_{j}\right\},\;A_{ij}=B_{i}\cap C_{j},\\ \; & b_{i}=\mu \left(B_{i}\right),\;c_{j}:=\mu \left(C_{j}\right),\;a_{ij}=\mu \left(A_{ij}\right). \end{array}$$

Moreover, $C_{i}\cap C_{j}=\emptyset$ and $B_{i}\cap B_{j}=\emptyset$ for every 1≤i≠j≤n; thus, $A_{ij}\cap A_{kl}=\emptyset$ for every ordered pair (i,j)≠(k,l). For obvious reasons, $B_{i}=\cup_{j=1}^{n}A_{ij}$ for each 1≤i≤n and $C_{j}=\cup_{i=1}^{n}A_{ij}$ for each 1≤j≤n, and $\Gamma =\cup_{i,j}A_{ij}$. So, we have $\sum_{i,j}a_{ij}=1$ and for every 1≤i,j≤n,

$$\begin{array}{c} b_{i}=\mu \left(B_{i}\right)=\mu \left(\overset{n}{\underset{j=1}{⋃}}A_{ij}\right)=\sum_{j=1}\mu \left(A_{ij}\right)=\sum_{j=1}^{n}a_{ij}, \end{array}$$

and

$$\begin{array}{c} c_{j}=\mu \left(C_{j}\right)=\mu \left(\cup_{i=1}^{n}A_{ij}\right)=\sum_{i=1}\mu \left(A_{ij}\right)=\sum_{i=1}^{n}a_{ij}. \end{array}$$

With the use of Lemma A1, we have

$$\begin{array}{c} \sum_{i=1}^{n}{\left(\sum_{j=1}^{n}a_{ij}\right)}^{2}+\sum_{j=1}^{n}{\left(\sum_{i=1}^{n}a_{ij}\right)}^{2}\leq 1+\sum_{i,j}a_{ij}^{2}. \end{array}$$

Therefore,

$$\begin{array}{c} \sum_{i=1}^{n}b_{i}^{2}+\sum_{j=1}^{n}c_{j}^{2}\leq 1+\sum_{i,j}a_{ij}^{2}. \end{array}$$

Consequently,

$$\begin{array}{c} \sum_{i=1}^{n}{\left(\mu \left(B_{i}\right)\right)}^{2}+\sum_{j=1}^{n}{\left(\mu \left(C_{j}\right)\right)}^{2}\leq 1+\sum_{i,j}{\left(\mu \left(A_{ij}\right)\right)}^{2}, \end{array}$$

and

$$\begin{array}{c} -\sum_{i,j}{\left(\mu \left(A_{ij}\right)\right)}^{2}\leq 1-\sum_{i=1}^{n}{\left(\mu \left(B_{i}\right)\right)}^{2}-\sum_{j=1}^{n}{\left(\mu \left(C_{j}\right)\right)}^{2}. \end{array}$$

Hence,

$$\begin{array}{c} 1-\sum_{i,j}{\left(\mu \left(A_{ij}\right)\right)}^{2}\leq \left(1-\sum_{i=1}^{n}{\left(\mu \left(B_{i}\right)\right)}^{2}\right)+\left(1-\sum_{j=1}^{n}{\left(\mu \left(C_{j}\right)\right)}^{2}\right), \end{array}$$

it follows that $H_{\mu l}\left(X_{1},X_{2}\right)\leq H_{\mu l}\left(X_{1}\right)+H_{\mu l}\left(X_{2}\right).$

Now, we prove the left-hand inequality. Since

$$\begin{array}{c} b_{i}=\mu \left(B_{i}\right)=\mu \left(\overset{n}{\underset{j=1}{⋃}}A_{ij}\right)=\sum_{j=1}\mu \left(A_{ij}\right)=\sum_{j=1}^{n}a_{ij} \end{array}$$

for every 1≤i≤n, $b_{i}^{2}={\left(\sum_{j=1}^{n}a_{ij}\right)}^{2}\geq \sum_{j=1}^{n}a_{ij}^{2}$. Therefore,

$$\begin{array}{c} {\left(\mu \left(B_{i}\right)\right)}^{2}\geq \sum_{j=1}^{n}{\left(\mu \left(A_{ij}\right)\right)}^{2}, \end{array}$$

and, thus,

$$\begin{array}{c} \sum_{i=1}^{n}{\left(\mu \left(B_{i}\right)\right)}^{2}\geq \sum_{i=1}^{n}\sum_{j=1}^{n}{\left(\mu \left(A_{ij}\right)\right)}^{2}. \end{array}$$

So, $H_{\mu l}\left(X_{1}\right)\leq H_{\mu l}\left(X_{1},X_{2}\right)$.

Similarly, $H_{\mu l}\left(X_{2}\right)\leq H_{\mu l}\left(X_{1},X_{2}\right)$. Consequently,

$$\begin{array}{c} \max\left\{H_{\mu l}\left(X_{1}\right),H_{\mu l}\left(X_{2}\right)\right\}\leq H_{\mu l}\left(X_{1},X_{2}\right). \end{array}$$

□

**Corollary** **1.** *If X is an information source, then*

$$\begin{array}{c} \max\left\{H_{\mu l}\left(X_{i}\right):1\leq i\leq k\right\}\leq H_{\mu l}\left(X_{1},\ldots ,X_{k}\right)\leq \sum_{i=1}^{k}H_{\mu l}\left(X_{i}\right),\;\left(\forall k\in N\right). \end{array}$$

**Proof.**  This follows from Theorem 3. □

**Definition** **4.** *The logical metric permutation entropy of order m of source $X=\{X_{0},X_{1},\ldots \}$ defined by*

$$\begin{array}{c} H_{\mu l}^{★}\left(X_{0}^{m-1}\right)=H_{\mu l}\left(R_{0}^{m-1}\right)=1-\sum_{r_{0},\ldots ,r_{m-1}}{\left(p\left(r_{0}^{m-1}\right)\right)}^{2}. \end{array}$$

**Lemma** **2.** *For a S.P, X, the sequence of ${\left\{H_{\mu l}\left(X_{1}^{m}\right)\right\}}_{m}$ increases. Thus, $\lim_{m\to \infty }H_{\mu l}\left(X_{1}^{m}\right)$ exists.*

**Proof.**  According to (1),

$$\begin{array}{c} p\left(x_{1},\ldots ,x_{m}\right)=\sum_{x_{m+1}}p\left(x_{1},\ldots ,x_{m},x_{m+1}\right) \end{array}$$

for every m∈N. Therefore,

$$\begin{array}{cc} {\left(p\left(x_{1},\ldots ,x_{m}\right)\right)}^{2} & ={\left(\sum_{x_{m+1}}p\left(x_{1},\ldots ,x_{m},x_{m+1}\right)\right)}^{2}\\ \; & \leq \sum_{x_{m+1}}{\left(p\left(x_{1},\ldots ,x_{m},x_{m+1}\right)\right)}^{2}, \end{array}$$

and

$$\begin{array}{c} \sum_{x_{1}^{m}}{\left(p\left(x_{1},\ldots ,x_{m}\right)\right)}^{2}\leq \sum_{x_{1}^{m+1}}{\left(p\left(x_{1},\ldots ,x_{m},x_{m+1}\right)\right)}^{2}. \end{array}$$

This means that

$$\begin{array}{cc} H_{\mu l}\left(X_{1}^{m}\right) & =1-\sum_{x_{1}^{m}}{\left(p\left(x_{1},\ldots ,x_{m}\right)\right)}^{2}\\ \; & \geq 1-\sum_{x_{1}^{m+1}}{\left(p\left(x_{1},\ldots ,x_{m},x_{m+1}\right)\right)}^{2}=H_{\mu l}\left(X_{1}^{m+1}\right). \end{array}$$

□

**Definition** **5.** *The logical Shannon entropy of source $X=\{X_{1},X_{2},\ldots \}$ is defined by*

$$\begin{array}{c} h_{\mu l}\left(X\right)=\lim_{m\to \infty }\left(H_{\mu l}\left(X_{1}^{m}\right)\right). \end{array}$$

**Definition** **6.** *The logical metric permutation entropy of source $X=\{X_{0},X_{1},\ldots \}$ is defined by*

$$\begin{array}{c} h_{\mu l}^{★}\left(X\right)=\lim_{m\to \infty }H_{\mu l}^{★}\left(X_{0}^{m-1}\right). \end{array}$$

**Remark** **1.** *Let m be a positive integer number. Then $0\leq H_{\mu l}\left(X_{1}^{m}\right)\leq 1$ and $0\leq h_{\mu l}\left(X\right)\leq 1$.*

**Lemma** **3.** *Let $X=(X_{1},X_{1},X_{1},\ldots )$ be an information source. Then the following holds:*

*1.* 
$H_{\mu l}\left(\underset{m\;times}{\underset{︸}{X_{1},X_{1},\ldots ,X_{1}}}\right)=H_{\mu l}\left(X_{1}\right),$ *for every* m∈N *.*
*2.*  *$h_{\mu l}\left(X\right)=H_{\mu l}\left(X_{1}\right)$.*

**Proof.** 

1. If $X=(X_{1},X_{1},X_{1},\ldots )$, then
  $$\begin{array}{c} p\left(x_{1},x_{2},\ldots ,x_{m}\right)=\left\{\begin{array}{cc} p(x_{1}) & x_{1}=x_{2}=\ldots =x_{m}\\ 0 & x_{i}\neq x_{j},\;\mathrm{for}\;\mathrm{some}\;1\leq i\neq j\leq m. \end{array}\right. \end{array}$$
  Hence,
  (3) $$\begin{array}{cc} H_{\mu l}\left(X_{1},\ldots ,X_{m}\right) & =\sum_{x_{1},\ldots ,x_{m}\in A}p\left(x_{1},\ldots ,x_{m}\right)(1-p(x_{1},\ldots ,x_{m}))\\ \; & =\sum_{x_{1}\in A}p\left(x_{1}\right)\left(1-p\left(x_{1}\right)\right)\\ \; & =H_{\mu l}\left(X_{1}\right). \end{array}$$
2. We derive from (3) that
  $$\begin{array}{cc} h_{\mu l}\left(X\right) & =\lim_{m\to \infty }H_{\mu l}\left(X_{1},\ldots ,X_{m}\right)\\ \; & =\lim_{m\to \infty }H_{\mu l}\left(X_{1},\ldots ,X_{1}\right)\\ \; & =\lim_{m\to \infty }H_{\mu l}\left(X_{1}\right)=H_{\mu l}\left(X_{1}\right). \end{array}$$

□

**Theorem** **4.** *Suppose that X represents an information source on *Γ* with the discrete finite state space $A=\{a_{1},\ldots ,a_{n}\}$.*

*1.*  *If $\zeta_{m}=\min_{x_{1}^{m}\in A}\left\{p\left(x_{1}^{m}\right)\right\}$ and $\eta_{m}=\max_{x_{1}^{m}\in A}\left\{p\left(x_{1}^{m}\right)\right\}$, then*
(4) $$\begin{array}{cc} 0\leq \Delta \left(\zeta_{m},\eta_{m}\right)\leq \frac{n^{m}-1}{n^{m}}-H_{\mu l}\left(x_{1}^{m}\right) & \leq n^{m}\Delta \left(\zeta_{m},\eta_{m}\right), \end{array}$$
*2.*  *
$\lim_{m\to \infty }\Delta \left(\zeta_{m},\eta_{m}\right)\leq 1-h_{\mu l}\left(X\right)\leq \lim_{m\to \infty }n^{m}\Delta \left(\zeta_{m},\eta_{m}\right).$
*

**Proof.** 

1. The result follows from Theorem 2.
2. Taking the limit as m→∞ in (4), consequently (2) holds.

□

**Lemma** **4.** *Let X represent an information source on *Γ* with the discrete finite state space $A=\{a_{1},\ldots ,a_{n}\}$, then $0\leq H_{\mu l}\left(X_{1}^{m}\right)\leq \frac{n^{m}-1}{n^{m}}$, and equality holds if and only if $p\left(x_{1}^{m}\right)=p\left(t_{1}^{m}\right)$ for every $x_{1}^{m},t_{1}^{m}\in A^{m}$.*

**Proof.**  By Theorem 4, $0\leq H_{\mu l}\left(X_{1}^{m}\right)\leq \frac{n^{m}-1}{n^{m}}$. If $H_{\mu l}\left(X_{1}^{m}\right)=\frac{n^{m}-1}{n^{m}}$, then by the use of Theorem 4 we obtain $\Delta \left(\zeta_{m},\eta_{m}\right)=\frac{{\left(\zeta_{m}-\eta_{m}\right)}^{2}}{4}=0$. Hence $\zeta_{m}=\eta_{m}$. Therefore $\max_{x_{1}^{m}\in A}\left\{p\left(x_{1}^{m}\right)\right\}$$=\min_{x_{1}^{m}\in A}\left\{p\left(x_{1}^{m}\right)\right\}$. Thus $p\left(x_{1}^{m}\right)=p\left(t_{1}^{m}\right)$ for every $x_{1}^{m},t_{1}^{m}\in A^{m}$. On the other hand if $p\left(x_{1}^{m}\right)=p\left(t_{1}^{m}\right)$ for every $x_{1}^{m},t_{1}^{m}\in A^{m}$, then $\zeta_{m}=\eta_{m}$. Therefore $\Delta (\zeta_{m},\eta_{m})=0$ and by Theorem 4 has $H_{\mu l}\left(X_{1}^{m}\right)-\frac{n^{m}-1}{n^{m}}=0$ and thus $H_{\mu l}\left(X_{1}^{m}\right)=\frac{n^{m}-1}{n^{m}}$. □

**Definition** **7.** *Let p(x)≠0, the conditional probability function defined by $p\left(y|x\right):=\frac{p(x,y)}{p(x)}$. In general, for $p(x_{1},\ldots ,x_{n})\neq 0$, the conditional probability function is defined by $p\left(x_{1}|x_{2},\ldots ,x_{n+1}\right):=\frac{p(x_{1},x_{2},\ldots ,x_{n+1})}{p(x_{2},x_{3},\ldots ,x_{n})}$.*

**Lemma** **5.** *Let $x_{1},x_{2},\ldots ,x_{n+1}$ be a word. Then*

$$\begin{array}{c} p\left(x_{m+1},x_{m},\ldots ,x_{1}\right)=\prod_{i=1}^{m+1}p\left(x_{i}|x_{i-1},\ldots ,x_{1}\right), \end{array}$$

*where m∈N and $p\left(x_{1}|x_{0}\right):=p\left(x_{1}\right)$.*

**Proof.**  We prove the lemma by induction. If m=1, have $p\left(x_{1},x_{2}\right)=p\left(x_{1}\right)\times p\left(x_{1}|x_{2}\right)$. Thus, the statement is true for m=1. Now suppose the statement is true for m=k−1, we give reasons for m=k.

$$\begin{array}{cc} \prod_{i=1}^{k+1}p\left(x_{i}|x_{i-1},\ldots ,x_{1}\right) & =\prod_{i=1}^{k}p\left(x_{i}|x_{i-1},\ldots ,x_{1}\right)\times p\left(x_{k+1}|x_{k},\ldots ,x_{1}\right)\\ \; & =p\left(x_{k},x_{k-1},\ldots ,x_{1}\right)\times p\left(x_{k+1}|x_{k},\ldots ,x_{1}\right)\\ \; & =p\left(x_{k},x_{k-1},\ldots ,x_{1}\right)\times \frac{p(x_{k+1},x_{k},\ldots ,x_{1})}{p(x_{k},x_{k-1},\ldots ,x_{1})}\\ \; & =p(x_{k+1},x_{k},\ldots ,x_{1}), \end{array}$$

which completes the proof. □

**Definition** **8.** *Let $X_{1}$ and $X_{2}$ be two random variables on *Γ*. We define the conditional logical entropy of $X_{2}$ given $X_{1}$ by*

$$\begin{array}{c} H_{\mu l}\left(X_{2}|X_{1}\right):=\sum_{x_{1},x_{2}}{\left(p\left(x_{1}\right)\right)}^{2}\left(p\left(x_{2}\right)-{\left(p\left(x_{2}|x_{1}\right)\right)}^{2}\right). \end{array}$$

*Note: if $p(x_{1})=0$, define ${\left(p\left(x_{1}\right)\right)}^{2}(p\left(x_{2}\right)-{\left(p\left(x_{2}|x_{1}\right)\right)}^{2}=0$.*

**Definition** **9.** *Suppose $X_{1},X_{2},\ldots ,X_{m}$ are m random variables on *Γ*. Define the conditional logical entropy of $X_{m}$ given $X_{1},\ldots ,X_{m-1}$ by*

$$\begin{array}{cc} H_{\mu l}\left(X_{m}|X_{m-1},\ldots ,X_{2},X_{1}\right): & =\sum_{x_{1}^{m}}{\left(p\left(x_{m-1},\ldots ,x_{2},x_{1}\right)\right)}^{2}\\ \; & [p\left(x_{m}\right)-{\left(p\left(x_{m}|x_{m-1},\ldots ,x_{1}\right)\right)}^{2}]. \end{array}$$

**Lemma** **6.** *Suppose $X_{1},X_{2},\ldots ,X_{m}$ are m random variables on Γ, then*

$$\begin{array}{cc} \; & H_{\mu l}\left(X_{m}|X_{m-1},\ldots ,X_{2},X_{1}\right)\\ \; & =\sum_{x_{1}^{m-1}}{\left(p\left(x_{m-1},\ldots ,x_{2},x_{1}\right)\right)}^{2}-\sum_{x_{1}^{m}}{\left(p\left(x_{m},\ldots ,x_{2},x_{1}\right)\right)}^{2}\\ \; & =H_{\mu l}\left(X_{m},X_{m-1},\ldots ,X_{2},X_{1}\right)-H_{\mu l}\left(X_{m-1},\ldots ,X_{2},X_{1}\right). \end{array}$$

**Proof.**  According to Definition 9, we obtain

$$\begin{array}{cc} \; & H_{\mu l}\left(X_{n}|X_{m-1},\ldots ,X_{2},X_{1}\right)\\ \; & =\sum_{x_{1}^{m}}{\left(p\left(x_{m-1},\ldots ,x_{2},x_{1}\right)\right)}^{2}\left(p\left(x_{m}\right)-{\left(p\left(x_{m}|x_{m-1},\ldots ,x_{1}\right)\right)}^{2}\right)\\ \; & =\sum_{x_{1}^{m}}p\left(x_{m-1},\ldots ,x_{2},x_{1}\right){)}^{2}p\left(x_{m}\right)\\ \; & -\sum_{x_{1}^{m}}p\left(x_{m-1},\ldots ,x_{2},x_{1}\right){)}^{2}{\left(p\left(x_{m}|x_{m-1},\ldots ,x_{1}\right)\right)}^{2}\\ \; & ={\left(\sum_{x_{1}^{m-1}}p\left(x_{m-1},\ldots ,x_{2},x_{1}\right)\right)}^{2})\left(\sum_{x_{m}}p\left(x_{m}\right)\right)\\ \; & -\sum_{x_{1}^{m}}p\left(x_{m-1},\ldots ,x_{2},x_{1}\right){)}^{2}{\left(\frac{p(x_{m},x_{m-1},\ldots ,x_{1})}{p(x_{m-1},\ldots ,x_{1})}\right)}^{2}\\ \; & =\sum_{x_{1}^{m-1}}{\left(p\left(x_{m-1},\ldots ,x_{2},x_{1}\right)\right)}^{2}-\sum_{x_{1}^{m}}{\left(p\left(x_{m},\ldots ,x_{2},x_{1}\right)\right)}^{2}. \end{array}$$

□

**Lemma** **7.** *Let X be a stationary finite space S.P, then*

(5) $$\begin{array}{c} \sum_{x_{2}^{n}}{\left(p\left(x_{n},\ldots ,x_{2}\right)\right)}^{2}=\sum_{x_{1}^{n-1}}{\left(p\left(x_{n-1},\ldots ,x_{2},x_{1}\right)\right)}^{2}. \end{array}$$

**Proof.**  Since X is stationary,

$$\begin{array}{cc} \sum_{x_{2}^{n}}{\left(p\left(x_{n},\ldots ,x_{2}\right)\right)}^{2} & =\sum_{x_{2}^{n}}{\left(\mu \left(\left\{\gamma \in \Gamma :X_{n}\left(\gamma \right)=x_{n},\ldots ,X_{2}\left(\gamma \right)=x_{2}\right\}\right)\right)}^{2}\\ \; & =\sum_{x_{2}^{n}}{\left(\mu \left(\left\{\gamma \in \Gamma :X_{n-1}\left(\gamma \right)=x_{n},\ldots ,X_{1}\left(\gamma \right)=x_{2}\right\}\right)\right)}^{2}\\ \; & =\sum_{x_{1}^{n-1}}{\left(p\left(x_{n-1},\ldots ,x_{2},x_{1}\right)\right)}^{2}, \end{array}$$

which yields (5). □

**Theorem** **5.** *Let X be a stationary finite space S.P, with discrete finite state space $A=\{a_{1},\ldots ,a_{n}\}$. Then the sequence of conditional logical entropies $H_{\mu l}\left(X_{m}|X_{m-1},\ldots ,X_{1}\right)$ decreases.*

**Proof.**  Under the notation of Definition 3, define

$$\begin{array}{c} \left\{A_{i_{1}i_{2}\ldots i_{m}}:1\leq i_{1},i_{2},\ldots ,i_{m}\leq n\right\}=\left\{D_{1},D_{2},\ldots ,D_{M}\right\}, \end{array}$$

and $\mu \left(D_{r}\right)=d_{r}$ where $M=n^{m}$. Furthermore, assume that

$$\begin{array}{c} D_{ij}=D_{i}⋂\left\{\gamma \in \Gamma :x_{j}\left(\gamma \right)=a_{j}\right\},\;\mu \left(D_{ij}\right)=d_{ij}, \end{array}$$

$$\begin{array}{c} D_{ijk}=D_{ij}⋂\left\{\gamma \in \Gamma :x_{k}\left(\gamma \right)=a_{k}\right\},\;\mu \left(D_{ijk}\right)=d_{ijk}, \end{array}$$

where 1≤i≤M and 1≤j,k≤n. It is easy to see that $D_{i}\cap D_{j}=\emptyset$ for every 1≤i≠j≤n, and $D_{ij}\cap D_{rs}=\emptyset$ for every ordered pair (i,j)≤(r,s). Therefore, $D_{ijk}\cap D_{rst}=\emptyset$ for every (i,j,k)≠(r,s,t). For obvious reasons, $D_{i}=\cup_{j=1}^{n}D_{ij}$ for each 1≤i≤n, $D_{ij}=\cup_{k=1}^{n}D_{ijk}$ for every 1≤i,j≤n and $\Gamma =\cup_{i,j,k}D_{ijk}$. Consequently, $\sum_{i,j,k}d_{ijk}=1$ and

$$\begin{array}{c} d_{i}=\mu \left(D_{i}\right)=\mu \left(\overset{n}{\underset{j=1}{⋃}}D_{ij}\right)=\sum_{j=1}^{n}\mu \left(D_{ij}\right)=\sum_{j=1}^{n}d_{ij} \end{array}$$

and

$$\begin{array}{c} d_{ij}=\mu \left(D_{ij}\right)=\mu \left(\overset{n}{\underset{k=1}{⋃}}D_{ijk}\right)=\sum_{k=1}^{n}\mu \left(D_{ijk}\right)=\sum_{k=1}^{n}d_{ijk} \end{array}$$

for every 1≤j≤M,1≤i≤n.

Using Theorem A1 and Lemma 7, we deduce that

$$\begin{array}{cc} \; & \sum_{x_{1}^{m+2}}{\left(p\left(x_{m+2},\ldots ,x_{2},x_{1}\right)\right)}^{2}=\sum_{i=1}^{M}\sum_{j=1}^{n}\sum_{k=1}^{n}d_{ijk}^{2}=\sum_{i,j,k}d_{ijk}^{2}\\ \; & \sum_{x_{1}^{m+1}}{\left(p\left(x_{m+1},\ldots ,x_{2},x_{1}\right)\right)}^{2}=\sum_{i=1}^{M}\sum_{j=1}^{n}d_{ij}^{2}=\sum_{i,j}d_{ij}^{2}=\sum_{i,j}{\left(\sum_{k=1}^{n}d_{ijk}\right)}^{2}\\ \; & \sum_{x_{1}^{m}}{\left(p\left(x_{m},\ldots ,x_{2},x_{1}\right)\right)}^{2}=\sum_{i=1}^{M}d_{i}^{2}=\sum_{i=1}^{M}{\left(\sum_{j,k=1}^{n}d_{ijk}\right)}^{2}\\ \; & \sum_{x_{2}^{m+2}}{\left(p\left(x_{m+2},\ldots ,x_{2}\right)\right)}^{2}=\sum_{x_{1}^{m+1}}{\left(p\left(x_{m+1},\ldots ,x_{2},x_{1}\right)\right)}^{2}=\sum_{i,j}d_{ij}^{2}. \end{array}$$

With the use of Theorem A1, we obtain

$$\begin{array}{cc} \; & H_{\mu l}\left(X_{m+2}|X_{m+1},\ldots ,X_{2},X_{1}\right)\\ \; & =\sum_{x_{1}^{m+1}}{\left(p\left(x_{m+1},\ldots ,x_{2},x_{1}\right)\right)}^{2}-\sum_{x_{1}^{m+2}}{\left(p\left(x_{m+2},\ldots ,x_{2},x_{1}\right)\right)}^{2}.\\ \; & =\sum_{i,j,k}d_{ijk}^{2}-\sum_{i,j}{\left(\sum_{k=1}^{n}d_{ijk}\right)}^{2}\\ \; & \geq \sum_{i,j}{\left(\sum_{k=1}^{n}d_{ijk}\right)}^{2}-\sum_{i=1}^{M}{\left(\sum_{j,k=1}^{n}d_{ijk}\right)}^{2}\\ \; & =\sum_{x_{1}^{m}}{\left(p\left(x_{m},\ldots ,x_{2},x_{1}\right)\right)}^{2}-\sum_{x_{1}^{m+1}}{\left(p\left(x_{m+1},\ldots ,x_{2},x_{1}\right)\right)}^{2}\\ \; & =H_{\mu l}\left(X_{m+1}|X_{m},\ldots ,X_{2},X_{1}\right), \end{array}$$

this means that the sequence of conditional logical entropies

$$\begin{array}{c} H_{\mu l}\left(X_{m}|X_{m-1},\ldots ,X_{1}\right) \end{array}$$

is decreasing, so

$$\begin{array}{c} 0\leq \ldots \leq H_{\mu l}\left(X_{m+1}|X_{m},\ldots ,X_{1}\right)\leq H_{\mu l}\left(X_{m}|X_{m-1},\ldots ,X_{1}\right)\leq \ldots \leq H_{\mu l}\left(X_{1}\right). \end{array}$$

□

**Corollary** **2.** *Let $X=(X_{1},X_{2},X_{3},\ldots )$ be a source. Then the limit $\lim_{n\to \infty }H_{\mu l}\left(X_{n}|X_{n-1},\ldots ,X_{1}\right)$ exists.*

**Lemma** **8.** *Let $X={\left(X_{m}\right)}_{m=1}^{\infty }$ be a stationary finite space S.P. Then*

$$\begin{array}{cc} \sum_{x_{2}^{m+1}}{\left(p\left(x_{m+1}|x_{m},\ldots ,x_{2}\right)\right)}^{2} & =\sum_{x_{1}^{m}}{\left(p\left(x_{m}|x_{m-1},\ldots ,x_{1}\right)\right)}^{2}. \end{array}$$

**Proof.**  Since X is stationary,

$$\begin{array}{cc} \; & \sum_{x_{2}^{m+1}}{\left(p\left(x_{m+1}|x_{m},\ldots ,x_{2}\right)\right)}^{2}\\ \; & =\sum_{x_{2}^{m+1}}{\left(\frac{p(x_{m+1},x_{m},\ldots ,x_{2})}{p(x_{m},\ldots ,x_{2})}\right)}^{2}\\ \; & =\sum_{x_{2}^{m+1}}{\left(\frac{\mu (\left\{\gamma \in \Gamma :x_{m+1}\left(\gamma \right)=x_{m+1},\ldots ,x_{2}\left(\Gamma \right)=x_{2}\right\})}{\mu (\left\{\Gamma \in \Gamma :x_{m}\left(\Gamma \right)=x_{m},\ldots ,x_{2}\left(\gamma \right)=x_{2}\right\})}\right)}^{2}\\ \; & =\sum_{x_{2}^{m+1}}{\left(\frac{\mu (\left\{\gamma \in \Gamma :x_{m}\left(\gamma \right)=x_{m+1},\ldots ,x_{1}\left(\gamma \right)=x_{2}\right\})}{\mu (\left\{\gamma \in \Gamma :x_{m-1}\left(\gamma \right)=x_{m},\ldots ,x_{1}\left(\gamma \right)=x_{2}\right\})}\right)}^{2}\\ \; & =\sum_{x_{1}^{m}}{\left(p\left(x_{m}|x_{m-1},\ldots ,x_{1}\right)\right)}^{2}, \end{array}$$

which completes the proof. □

**Theorem** **6.** *Let $X={\left(X_{n}\right)}_{n=1}^{\infty }$ be a stationary finite space S.P. Then*

$$\begin{array}{c} H_{\mu l}\left(X_{m+1}|X_{m},\ldots ,X_{2}\right)=H_{\mu l}\left(X_{m}|X_{m-1},\ldots ,X_{1}\right) \end{array}$$

**Proof.**  According to Lemma 7,

$$\begin{array}{cc} H_{\mu l}\left(X_{m+1}|X_{m},\ldots ,X_{2}\right) & =\sum_{x_{2}^{m}}{\left(p\left(x_{m},\ldots ,x_{2}\right)\right)}^{2}-\sum_{x_{2}^{m+1}}{\left(p\left(x_{m},\ldots ,x_{2}\right)\right)}^{2}\\ \; & =\sum_{x_{1}^{m-1}}{\left(p\left(x_{m-1},\ldots ,x_{1}\right)\right)}^{2}-\sum_{x_{1}^{m}}{\left(p\left(x_{m},\ldots ,x_{1}\right)\right)}^{2}\\ \; & =H_{\mu l}\left(X_{m}|X_{m-1},\ldots ,X_{2},X_{1}\right). \end{array}$$

Theorem 6 is thus proved. □

**Theorem** **7.** *Let $X_{1}$ and $X_{2}$ be two random variables on *Γ*. Then the following hold:*

*1.* 
$H_{\mu l}\left(X_{2}|X_{1}\right)=H_{\mu l}\left(X_{1},X_{2}\right)-H_{\mu l}\left(X_{1}\right).$

*2.*  *$H_{\mu l}\left(X_{2}|X_{1}\right)+H_{\mu l}\left(X_{1}\right)=H_{\mu l}\left(X_{1}|X_{2}\right)+H_{\mu l}\left(X_{2}\right)$.*

**Proof.** 

1. Using the definition of condition logical entropy, we deduce
  $$\begin{array}{cc} H_{\mu l}\left(X_{2}|X_{1}\right) & =\left(\sum_{x_{1}}{\left(p\left(x_{1}\right)\right)}^{2}\right)-\sum_{x_{1},x_{2}}{\left(p\left(x_{1},x_{2}\right)\right)}^{2}\\ \; & =\left(1-\sum_{x_{1},x_{2}}{\left(p\left(x_{1},x_{2}\right)\right)}^{2}\right)-\left(1-\sum_{x_{1}}{\left(p\left(x_{1}\right)\right)}^{2}\right)\\ \; & =H_{\mu l}\left(X_{1},X_{2}\right)-H_{\mu l}\left(X_{1}\right), \end{array}$$
  which completes the proof.
2. From the previous part, and since $H_{\mu l}\left(X_{1},X_{2}\right)=H_{\mu l}\left(X_{2},X_{1}\right)$, we have
  $$\begin{array}{cc} H_{\mu l}\left(X_{2}|X_{1}\right)+H_{\mu l}\left(X_{1}\right) & =H_{\mu l}\left(X_{1},X_{2}\right)\\ \; & =H_{\mu l}\left(X_{2},X_{1}\right)\\ \; & =H_{\mu l}\left(X_{1}|X_{2}\right)+H_{\mu l}\left(X_{2}\right). \end{array}$$

□

**Theorem** **8.** *Let $X=(X_{1},X_{1},X_{1},\ldots )$ be an information source. Then*

$$\begin{array}{c} H_{\mu l}\left(X_{1},\ldots ,X_{m}\right)=\sum_{i=1}^{m}H_{\mu l}\left(X_{i}|X_{i-1},\ldots ,X_{1}\right), \end{array}$$

*where $H_{\mu l}\left(X_{1}|X_{0}\right):=H_{\mu l}\left(X_{1}\right)$.*

**Proof.**  According to Lemma 6, we obtain

$$\begin{array}{cc} \; & \sum_{i=1}^{m}H_{\mu l}\left(X_{i}|X_{i-1},\ldots ,X_{1}\right)\\ \; & =H_{\mu l}\left(X_{1}\right)+\sum_{i=2}^{m}\left(H_{\mu l}\left(X_{i},\ldots ,X_{1}\right)-H_{\mu l}\left(X_{i-1},\ldots ,X_{1}\right)\right)\\ \; & =H_{\mu l}\left(X_{1},\ldots ,X_{m}\right), \end{array}$$

hence the theorem is proven. □

**Theorem** **9.** *Let $X=(X_{1},X_{2},X_{3},\ldots )$ be an information source. Then*

$$\begin{array}{c} h_{\mu l}\left(X\right)=\sum_{i=1}^{\infty }H_{\mu l}\left(X_{i}|X_{i-1},\ldots ,X_{1}\right). \end{array}$$

**Proof.**  By the use of Theorem 8, we obtain

$$\begin{array}{c} h_{\mu l}\left(X\right)=\lim_{n\to \infty }\sum_{i=1}^{n}H_{\mu l}\left(X_{i}|X_{i-1},\ldots ,X_{1}\right)=\sum_{i=1}^{\infty }H_{\mu l}\left(X_{i}|X_{i-1},\ldots ,X_{1}\right), \end{array}$$

which completes the proof. □

**Definition** **10.** *An independent information source, $X=(X_{1},X_{2},X_{3},\ldots )$, is a source with the following property*

$$\begin{array}{c} p\left(x_{1},x_{2},\ldots ,x_{m}\right)=\prod_{i=1}^{m}p\left(x_{i}\right) \end{array}$$

*for all $x_{1}^{m}$.*

**Theorem** **10.** *Let $X=(X_{1},X_{2},X_{3},\ldots )$ be an independent information source. Then*

$$\begin{array}{c} H_{\mu l}\left(X_{m+1}|X_{m},\ldots ,X_{1}\right)=\left(1-H_{\mu l}\left(X_{m},\ldots ,X_{1}\right)\right)H_{\mu l}\left(X_{m+1}\right) \end{array}$$

*for every m∈N.*

**Proof.**  Since $X=(X_{1},X_{2},X_{3},\ldots )$ is an independent random variables, we have

(6) $$\begin{array}{cc} \; & H_{\mu l}\left(X_{m+1}|X_{m},\ldots ,X_{1}\right)\\ \; & =\sum_{x_{1}^{m+1}}{\left(p\left(x_{m},\ldots ,x_{1}\right)\right)}^{2}p\left(x_{m+1}\right)-\sum_{x_{1}^{m+1}}{\left(p\left(x_{m+1},\ldots ,x_{1}\right)\right)}^{2}\\ \; & =\sum_{x_{1}^{m+1}}{\left(p\left(x_{m},\ldots ,x_{1}\right)\right)}^{2}p\left(x_{m+1}\right)-\sum_{x_{1}^{m+1}}{\left(p\left(x_{m+1},\ldots ,x_{1}\right)\right)}^{2}\\ \; & =\sum_{x_{1}^{m+1}}{\left(p\left(x_{m},\ldots ,x_{1}\right)\right)}^{2}p\left(x_{m+1}\right)-\sum_{x_{1}^{m+1}}{\left(p\left(x_{m},\ldots ,x_{1}\right)\right)}^{2}{\left(p\left(x_{m+1}\right)\right)}^{2}\\ \; & =\sum_{x_{1}^{m+1}}{\left(p\left(x_{m},\ldots ,x_{1}\right)\right)}^{2}\left(p\left(x_{m+1}\right)-{\left(p\left(x_{m+1}\right)\right)}^{2}\right)\\ \; & =\left(\sum_{x_{1}^{m}}{\left(p\left(x_{m},\ldots ,x_{1}\right)\right)}^{2}\right)\left(\sum_{x_{m+1}}\left(p\left(x_{m+1}\right)-{\left(p\left(x_{m+1}\right)\right)}^{2}\right)\right)\\ \; & =\left(1-H_{\mu l}\left(X_{m},\ldots ,X_{1}\right)\right)H_{\mu l}\left(X_{m+1}\right). \end{array}$$

The result follows from (6). □

**Theorem** **11.** *Suppose that $X=(X_{1},X_{2},X_{3},\ldots )$ is an independent information source and $\lim_{n}H_{\mu l}\left(X_{n}\right)\neq 0$. Then $h_{\mu l}\left(X\right)=1$.*

**Proof.**  In view of Theorem 10 and Lemma A2, we conclude that

$$\begin{array}{cc} \lim_{n\to \infty }H_{\mu l}\left(X_{n+1}|X_{n},\ldots ,X_{1}\right) & =\lim_{n\to \infty }\left(1-H_{\mu l}\left(X_{n},\ldots ,X_{1}\right)\right)H_{\mu l}\left(X_{n+1}\right)\\ \; & =\lim_{n}\left(1-H_{\mu l}\left(X_{n},\ldots ,X_{1}\right)\right)\times \lim_{n}H_{\mu l}\left(X_{n+1}\right)=0. \end{array}$$

Since $\lim_{n}H_{\mu l}\left(X_{n}\right)\neq 0$, $\lim_{n}\left(1-H_{\mu l}\left(X_{n},\ldots ,X_{1}\right)\right)=0$. Hence,

$$\begin{array}{c} h_{\mu l}\left(X\right)=\lim_{n\to \infty }H_{\mu l}\left(X_{n},\ldots ,X_{1}\right)=1. \end{array}$$

□

**Theorem** **12.** *Let $X=(X_{1},X_{2},X_{3},\ldots )$ be an independent information source. Then*

$$\begin{array}{c} H_{\mu l}\left(X_{m},\ldots ,X_{1}\right)=1-\prod_{i=1}^{m}\left(1-H_{\mu l}\left(X_{i}\right)\right) \end{array}$$

*for every m∈N.*

**Proof.**  Since X is an independent source,

$$\begin{array}{cc} H_{\mu l}\left(X_{m},\ldots ,X_{1}\right) & =1-\sum_{x_{1},\ldots ,x_{m}}(p\left(x_{1},\ldots ,x_{m}\right){)}^{2}\\ \; & =1-\sum_{x_{1},\ldots ,x_{m}}{\left(\prod_{i=1}^{m}p\left(x_{i}\right)\right)}^{2}=1-\sum_{x_{1},\ldots ,x_{m}}{\left(\prod_{i=1}^{m}(p\left(x_{i}\right)\right)}^{2})\\ \; & =1-\prod_{i=1}^{m}\left(\sum_{x_{i}}{\left(p\left(x_{i}\right)\right)}^{2}\right)=1-\prod_{i=1}^{m}\left(1-H_{\mu l}\left(X_{i}\right)\right), \end{array}$$

which is the desired result. □

**Theorem** **13.** *If $X=(X_{1},X_{2},X_{3},\ldots )$ is an independent information source, then*

*1.* 
$\lim_{n\to \infty }\prod_{i=1}^{n}\left(1-H_{\mu l}\left(X_{i}\right)\right)=1-h_{\mu l}\left(X\right).$

*2.*  *If there exists k∈N, such that $H_{\mu l}\left(X_{k}\right)=1$, then $h_{\mu l}\left(X\right)=1$.*

**Proof.** 

1. This follows from Theorem 12.
2. Let $H_{\mu l}\left(X_{k}\right)=1$ for some k∈N. Since $H_{\mu l}\left(X_{k}\right)=1$,
  $$\begin{array}{c} 1-h_{\mu l}\left(X\right)=\lim_{n\to \infty }\prod_{i=1}^{n}\left(1-H_{\mu l}\left(X_{i}\right)\right)=0. \end{array}$$

Hence, $h_{\mu l}\left(X\right)=1$. □

**Definition** **11.** *Let $X_{1}$ and $X_{2}$ be two random variables on *Γ*. Define the logical mutual information of $X_{2}$ and $X_{1}$ by*

$$\begin{array}{c} I_{\mu l}\left(X_{1},X_{2}\right):=H_{\mu l}\left(X_{1}\right)-H_{\mu l}\left(X_{1}|X_{2}\right). \end{array}$$

**Lemma** **9.** *Let $X_{1}$ and $X_{2}$ be two random variables on *Γ*. Then the following hold:*

*1.*  *$I_{\mu l}\left(X_{1},X_{2}\right)=H_{\mu l}\left(X_{2}\right)-H_{\mu l}\left(X_{2}|X_{1}\right)$.*
*2.*  *$I_{\mu l}\left(X_{1},X_{2}\right)=H_{\mu l}\left(X_{1}\right)+H_{\mu l}\left(X_{2}\right)-H_{\mu l}\left(X_{1},X_{2}\right)$.*
*3.*  *$I_{\mu l}\left(X_{1},X_{2}\right)=I_{\mu l}\left(X_{2},X_{1}\right)$.*
*4.*  *$I_{\mu l}\left(X_{1},X_{1}\right)=H_{\mu l}\left(X_{1}\right)$.*
*5.*  *If $X_{1}$ and $X_{2}$ are independent random variables, then*
$$\begin{array}{c} I_{\mu l}\left(X_{1},X_{2}\right)=H_{\mu l}\left(X_{1}\right)H_{\mu l}\left(X_{2}\right). \end{array}$$

**Proof.** 

1–3. follows from Definition 11 and Theorem 7.
4. According to Lemma 3, $H_{\mu l}\left(X_{1},X_{1}\right)=H_{\mu l}\left(X_{1}\right)$. Therefore,
  $$\begin{array}{cc} I_{\mu l}\left(X_{1},X_{1}\right) & =H_{\mu l}\left(X_{1}\right)+H_{\mu l}\left(X_{1}\right)-H_{\mu l}\left(X_{1},X_{1}\right)\\ \; & =2H_{\mu l}\left(X_{1}\right)-H_{\mu l}\left(X_{1}\right))=H_{\mu l}\left(X_{1}\right). \end{array}$$
5. It follows from Lemma 12 that
  $$\begin{array}{cc} H_{\mu l}\left(X_{1},X_{2}\right) & =1-\left(1-H_{\mu l}\left(X_{1}\right)\right)\left(1-H_{\mu l}\left(X_{2}\right)\right)\\ \; & =H_{\mu l}\left(X_{1}\right)+H_{\mu l}\left(X_{2}\right)-H_{\mu l}\left(X_{1}\right)H_{\mu l}\left(X_{2}\right). \end{array}$$

Hence, the result follows from 2. □

**Definition** **12.** *Let $X=(X_{1},X_{2},X_{3},\ldots )$ be an information source. Define the logical mutual information of $X_{1}$,…,$X_{m}$ by*

$$\begin{array}{c} I_{\mu l}\left(X_{1},\ldots ,X_{m}\right):=\sum_{i=1}^{m}H_{\mu l}\left(X_{i}\right)-H_{\mu l}\left(X_{1},\ldots ,X_{m}\right). \end{array}$$

**Lemma** **10.** *Let $X_{1}$ and $X_{2}$ be two random variables on *Γ*. Then*

$$\begin{array}{c} 0\leq H_{\mu l}\left(X_{2}|X_{1}\right)\leq H_{\mu l}\left(X_{2}\right). \end{array}$$

**Proof.**  It follows from Theorem 8 that

$$\begin{array}{c} H_{\mu l}\left(X_{1},X_{2}\right)=H_{\mu l}\left(X_{1}\right)+H_{\mu l}\left(X_{2}|X_{1}\right) \end{array}$$

and from Theorem 3 that $H_{\mu l}\left(X_{1},X_{2}\right)\leq H_{\mu l}\left(X_{1}\right)+H_{\mu l}\left(X_{2}\right)$. Hence,

$$\begin{array}{c} H_{\mu l}\left(X_{1}\right)+H_{\mu l}\left(X_{2}|X_{1}\right)\leq H_{\mu l}\left(X_{1}\right)+H_{\mu l}\left(X_{2}\right). \end{array}$$

This means that $H_{\mu l}\left(X_{2}|X_{1}\right)\leq H_{\mu l}\left(X_{2}\right).$ □

**Theorem** **14.** *Let $X_{1}$ and $X_{2}$ be two random variables on *Γ*. Then the following holds:*

$$\begin{array}{c} 0\leq I_{\mu l}\left(X_{1},X_{2}\right)\leq \min\left\{H_{\mu l}\left(X_{1}\right),H_{\mu l}\left(X_{1}\right)\right\}. \end{array}$$

**Proof.**  From Lemma 9, it follows that

$$\begin{array}{c} I_{\mu l}\left(X_{1},X_{2}\right)=H_{\mu l}\left(X_{1}\right)+H_{\mu l}\left(X_{2}\right)-H_{\mu l}\left(X_{1},X_{2}\right). \end{array}$$

Furthermore, Theorem 3 yields $H_{\mu l}\left(X_{2}\right)\leq H_{\mu l}\left(X_{1},X_{2}\right)$. Hence,

$$\begin{array}{cc} I_{\mu l}\left(X_{1},X_{2}\right) & =H_{\mu l}\left(X_{1}\right)+H_{\mu l}\left(X_{2}\right)-H_{\mu l}\left(X_{1},X_{2}\right)\\ \; & \leq H_{\mu l}\left(X_{1}\right)+H_{\mu l}\left(X_{1},X_{2}\right)-H_{\mu l}\left(X_{1},X_{2}\right)\\ \; & =H_{\mu l}\left(X_{1}\right). \end{array}$$

Similarly, $I_{\mu l}\left(X_{1},X_{2}\right)\leq H_{\mu l}\left(X_{2}\right)$; therefore,

$$\begin{array}{c} I_{\mu l}\left(X_{1},X_{2}\right)\leq \min\left\{H_{\mu l}\left(X_{1}\right),H_{\mu l}\left(X_{1}\right)\right\}. \end{array}$$

On the other hand, (2) yields

$$\begin{array}{c} H_{\mu l}\left(X_{2}\right)+H_{\mu l}\left(X_{2}\right)-H_{\mu l}\left(X_{1},X_{2}\right)\geq 0. \end{array}$$

Therefore, $I_{\mu l}\left(X_{1},X_{2}\right)\geq 0$ and, thus,

$$\begin{array}{c} 0\leq I_{\mu l}\left(X_{1},X_{2}\right)\leq \min\left\{H_{\mu l}\left(X_{1}\right),H_{\mu l}\left(X_{1}\right)\right\}. \end{array}$$

□

## 3. Logical Entropy of Maps

**Definition** **13.** *Let f:Γ⟶Γ be a measurable function and $\alpha =\{\alpha_{1},\ldots ,\alpha_{n}\}$ be a partition of *Γ*. The logical metric entropy of order m of f with respect to the partition α is defined by*

(7) $$\begin{array}{c} h_{\mu l,m}\left(f,\alpha \right)=1-\sum_{1\leq x_{0},\ldots ,x_{m}\leq n}(\mu {\left(\alpha_{x_{0}}\cap f^{-1}\left(\alpha_{x_{1}}\right)\cap \ldots \cap f^{-m}\left(\left(\alpha_{x_{m}}\right)\right)\right)}^{2}, \end{array}$$

*and the logical metric entropy of f with respect to the partition α is defined by*

(8) $$\begin{array}{c} h_{\mu l}\left(f,\alpha \right)=\lim_{m\to \infty }h_{\mu l,m}\left(f,\alpha \right). \end{array}$$

*The limits in (7) and (8) exist (see Theorem 15). The logical metric entropy of f is defined by $h_{\mu l}\left(f\right)=\sup_{\alpha }h_{\mu l}\left(f,\alpha \right)$.*

**Remark** **2.** *$0\leq h_{\mu l}\left(f\right)\leq 1$.*

Let *I* be an interval, h:I⟶I be a function and x∈I. For the finite orbit $\left\{h^{n}\left(x\right):0\leq n\leq L-1\right\}$, we say that *x* is of type ordinal *L*-pattern $\pi =\pi \left(x\right)=\left(\pi_{0},\ldots ,\pi_{L-1}\right)$ if

$$\begin{array}{c} h^{\pi_{0}}\left(x\right)<h^{\pi_{1}}\left(x\right)<\ldots <h^{\pi_{L-1}}\left(x\right). \end{array}$$

We denote $P_{\pi }$ the set of x∈I that are of type π.

**Definition** **14.** *The logical metric permutation entropy of order m of f is defined by*

$$\begin{array}{c} H_{\mu l,m}^{★}\left(f\right):=1-\sum_{\pi \in S_{m}}{\left(\mu \left(p_{\pi }\right)\right)}^{2}, \end{array}$$

*and the logical metric permutation entropy of f is defined by*

$$\begin{array}{c} h_{\mu l}^{*}\left(f\right):=\lim_{m\to \infty }H_{\mu l,m}^{★}\left(f\right)=1-\lim_{m\to \infty }\sum_{\pi \in S_{m}}{\left(\mu \left(p_{\pi }\right)\right)}^{2}. \end{array}$$

**Theorem** **15.** *Given A={0,1,…,n−1} with $X_{m}:\left[0,1\right]⟶A$, is defined as follows:*

$$\begin{array}{c} X_{m}\left(x\right)=i⟺f^{m}\left(x\right)\in \alpha_{i}. \end{array}$$

*Then $h_{\mu l}\left(f,\alpha \right)=h_{\mu l}\left(X\right)$ where X is a stationary process $\left(X_{0},X_{1},\ldots \right)$.*

**Proof.**  Since

$$\begin{array}{cc} H_{\mu l}\left(X_{m}\right) & =1-\sum_{i=0}^{n-1}{\left(\mu \left(\left\{x:f^{m}\left(x\right)\in \alpha_{i}\right\}\right)\right)}^{2}\\ \; & =1-\sum_{i=0}^{n-1}{\left(\mu \left(f^{-m}\alpha_{i}\right)\right)}^{2}, \end{array}$$

we have

$$\begin{array}{cc} p(x_{0},\ldots ,x_{m}) & =\mu (\left\{x:x_{0}\left(x\right)=x_{0},\ldots ,x_{m}\left(x\right)=x_{m}\right\})\\ \; & =\mu (\left\{x:x\in \alpha_{x_{0}},\ldots ,f^{m}\left(x\right)\in \alpha_{x_{m}}\right\})\\ \; & =\mu (\alpha_{x_{0}}⋂f^{-1}\left(\alpha_{x_{1}}\right)\cap \ldots ⋂f^{-m}\left(\left(\alpha_{x_{m}}\right)\right). \end{array}$$

Hence,

(9) $$\begin{array}{cc} H_{\mu l}\left(X_{0}^{m}\right) & =1-\sum_{x_{0}^{m}}(\mu {\left(\alpha_{x_{0}}⋂f^{-1}\left(\alpha_{x_{1}}\right)⋂\ldots ⋂f^{-m}\left(\left(\alpha_{x_{m}}\right)\right)\right)}^{2}, \end{array}$$

and so (9) implies that $h_{\mu l}\left(f,\alpha \right)=h_{\mu l}\left(X\right)$. □

## 4. Examples and Applications in Logistic and Tent Maps

**Figure 1:**
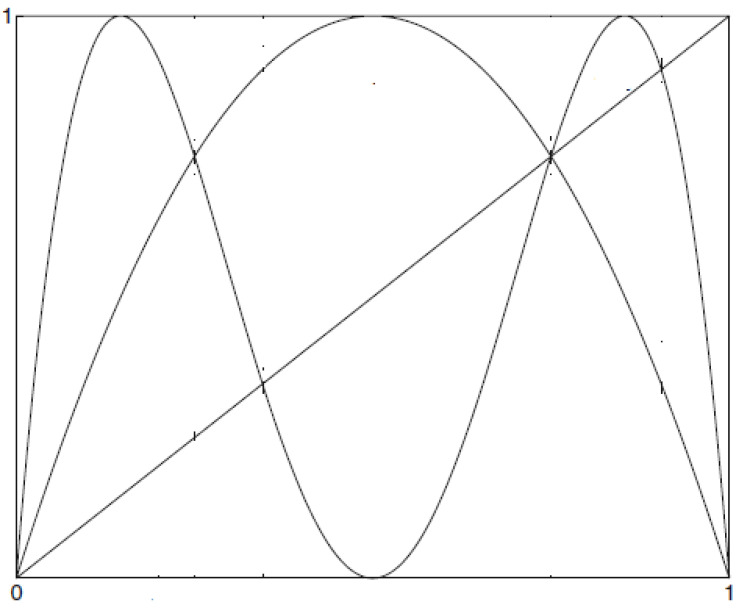
$x,g\left(x\right),\;\mathrm{and}\;g^{2}\left(x\right)$.

**Figure 2:**
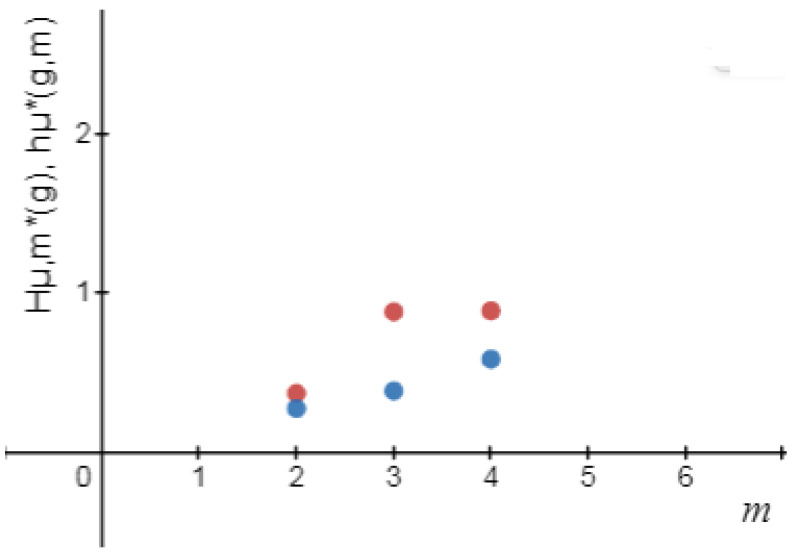
$H_{\mu l,m}^{★}\left(g\right),\;\mathrm{and}\;h_{\mu }^{★}\left(g,m\right)$.

**Example** **1.** *Let g(x)=4x(1−x):[0,1]⟶[0,1] be the logistic map (see Figure 1 and Figure 2 and Table 1). Then*

$$\begin{array}{cc} \; & p_{\left(0,1\right)}=\left(0,\frac{3}{4}\right),\;p_{\left(1,0\right)}=\left(\frac{3}{4},1\right),\\ \; & p_{\left(0,1,2\right)}=\left(0,\frac{1}{4}\right),\;p_{\left(0,2,1\right)}=\left(\frac{1}{4},\frac{5-\sqrt{5}}{8}\right),\;p_{\left(2,0,1\right)}=\left(\frac{5-\sqrt{5}}{8},\frac{3}{4}\right),\\ \; & p_{\left(1,0,2\right)}=\left(\frac{3}{4},\frac{5+\sqrt{5}}{8}\right),\;p_{\left(1,2,0\right)}=\left(\frac{5+\sqrt{5}}{8},1\right),\;p_{\left(2,1,0\right)}=\emptyset . \end{array}$$

*Therefore,*

$$\begin{array}{cc} \; & \sum_{\pi \in S_{2}}{\left(\mu \left(p_{\pi }\right)\right)}^{2}={\left(\frac{3}{4}\right)}^{2}+{\left(\frac{1}{4}\right)}^{2}=\frac{5}{8},\\ \; & H_{\mu l,2}^{★}\left(g\right)=1-\frac{5}{8}=0/375, \end{array}$$

$$\begin{array}{cc} \sum_{\pi \in S_{3}}{\left(\mu \left(p_{\pi }\right)\right)}^{2} & ={\left(\frac{1}{4}\right)}^{2}+{\left(\frac{3-\sqrt{5}}{8}\right)}^{2}+{\left(\frac{1+\sqrt{5}}{8}\right)}^{2}+{\left(\frac{\sqrt{5}-1}{8}\right)}^{2}+{\left(\frac{3-\sqrt{5}}{8}\right)}^{2},\\ \; & =\frac{17-6\sqrt{5}}{32} \end{array}$$

*and*

$$\begin{array}{c} H_{\mu l,3}^{★}\left(g\right)=1-\frac{17-6\sqrt{5}}{32}=\frac{15+6\sqrt{5}}{32}≃0/888. \end{array}$$

**Figure 3:**
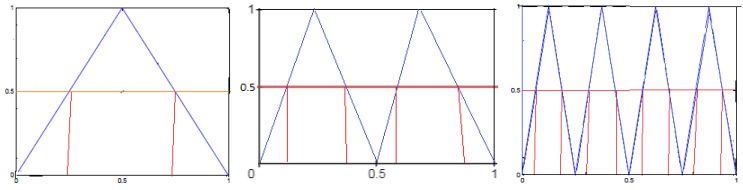
$\Lambda ,\Lambda^{2}\;\mathrm{and}\;\Lambda^{3}$.

**Figure 4:**
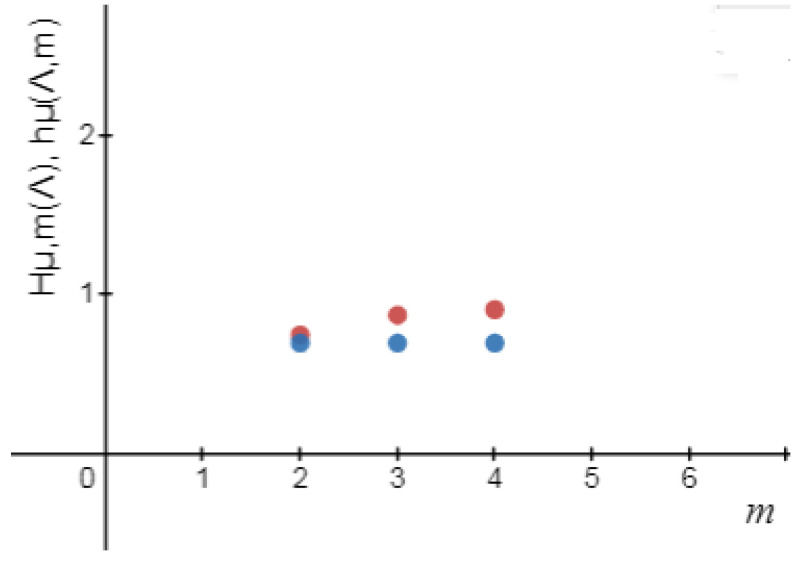
$H_{\mu l,m}\left(\Lambda \right),\;\mathrm{and}\;h_{\mu }\left(\Lambda ,m\right)$.

**Example** **2.** *Ref. [1] Let $\alpha =\{\alpha_{0}=\left[0,\frac{1}{2}\right],\alpha_{1}=\left(\frac{1}{2},1\right]\}$. We consider the tent map (see Figure 3 and Figure 4 and Table 2) Λ:[0,1]⟶[0,1] by*

$$\begin{array}{c} \Lambda \left(x\right)=\left\{\begin{array}{cc} 2x\; & 0\leq x\leq \frac{1}{2},\\ 2-2x\; & \frac{1}{2}\leq x\leq 1, \end{array}.\right. \end{array}$$

*Define $X_{m}:\left[0,1\right]⟶\left\{0,1\right\}$ via*

$$\begin{array}{c} X_{m}\left(x\right)=\left\{\begin{array}{cc} 0\; & \;\mathrm{if}\;\Lambda^{m}\left(x\right)\in \alpha_{0},\\ 1\; & \;\mathrm{if}\;\Lambda^{m}\left(x\right)\in \alpha_{1}, \end{array}\right. \end{array}$$

*for every m≥0. Let*

$$\begin{array}{cc} \; & \alpha_{00}=\left[0,\frac{1}{4}\right]=\left\{x\in \alpha_{0}:\Lambda \left(x\right)\in \alpha_{0}\right\},\alpha_{01}=\left(\frac{1}{4},\frac{1}{2}\right]=\left\{x\in \alpha_{0}:\Lambda \left(x\right)\in \alpha_{1}\right\},\\ \; & \;\alpha_{10}=\left[\frac{3}{4},1\right]=\left\{x\in \alpha_{1}:\Lambda \left(x\right)\in \alpha_{0}\right\},\alpha_{11}=\left(\frac{1}{2},\frac{3}{4}\right)=\left\{x\in \alpha_{1}:\Lambda \left(x\right)\in \alpha_{1}\right\}. \end{array}$$

*Given $\alpha_{i_{1}\ldots i_{m}}$, where m∈N, set*

$$\begin{array}{c} \alpha_{i_{1}\ldots i_{m}0}=\alpha_{i_{1}\ldots i_{m}}⋂\left\{x\in \left[0,1\right]:\Lambda^{m}\left(x\right)\in \alpha_{0}\right\}, \end{array}$$

$$\begin{array}{c} \alpha_{i_{1}\ldots i_{m}1}=\alpha_{i_{1}\ldots i_{m}}⋂\left\{x\in \left[0,1\right]:\Lambda^{m}\left(x\right)\in \alpha_{1}\right\}, \end{array}$$

*and*

$$\begin{array}{c} \alpha_{i_{0}i_{1}\ldots i_{m}}=\overset{m}{\underset{k=0}{⋂}}\Lambda^{-k}\alpha_{i_{k}}. \end{array}$$

*Therefore,*

$$\begin{array}{cc} \; & \alpha_{000}=\left[0,\frac{1}{8}\right],\;\alpha_{001}=\left(\frac{1}{8},\frac{1}{4}\right],\;\alpha_{010}=\left[\frac{3}{8},\frac{1}{2}\right],\;\alpha_{011}=\left(\frac{1}{4},\frac{3}{8}\right),\\ \; & \alpha_{100}=\left[\frac{7}{8},1\right],\;\alpha_{101}=\left[\frac{3}{4},\frac{7}{8}\right),\alpha_{110}=\left(\frac{1}{2},\frac{5}{8}\right],\;\alpha_{111}=\left(\frac{5}{8},\frac{3}{4}\right),\\ \; & \alpha_{0000}=\left[0,\frac{1}{16}\right],\;\alpha_{0001}=\left(\frac{1}{16},\frac{1}{8}\right],\;\alpha_{0010}=\left[\frac{3}{16},\frac{1}{4}\right],\;\alpha_{0011}=\left(\frac{1}{8},\frac{3}{16}\right),\\ \; & \alpha_{0100}=\left[\frac{7}{16},\frac{1}{2}\right],\;\alpha_{0101}=\left[\frac{3}{8},\frac{7}{16}\right),\;\alpha_{0110}=\left(\frac{1}{4},\frac{5}{16}\right],\;\alpha_{0111}=\left(\frac{5}{16},\frac{3}{8}\right),\\ \; & \alpha_{1000}=\left[\frac{15}{16},1\right],\;\alpha_{1000}=\left[\frac{7}{8},\frac{15}{16}\right),\;\alpha_{1010}=\left[\frac{3}{4},\frac{13}{16}\right],\;\alpha_{1011}=\left(\frac{13}{16},\frac{7}{8}\right),\\ \; & \alpha_{1100}=\left(\frac{1}{2},\frac{9}{16}\right],\;\alpha_{1101}=\left(\frac{9}{16},\frac{5}{8}\right],\alpha_{1110}=\left[\frac{11}{16},\frac{3}{4}\right),\;\alpha_{1111}=\left(\frac{5}{8},\frac{3}{4}\right). \end{array}$$

*The sets $\left\{\alpha_{i_{0}\ldots i_{m-1}}\right\}$ are identical to the binary sequences of 0, 1 in length m.*

*Hence, $\mu \left(\alpha_{i_{0}\ldots i_{m-1}}\right)=\frac{1}{2^{m}}$ and, thus,*

$$\begin{array}{cc} H_{\mu l}\left(X_{0}^{m-1}\right) & =1-\sum_{i_{0}\ldots i_{m-1}}{\left(\mu \left(\alpha_{i_{1}\ldots i_{m}}\right)\right)}^{2}=1-\sum_{i_{0}^{m-1}}{\left(\frac{1}{2^{m}}\right)}^{2}\\ \; & =1-2^{m}\times \frac{1}{2^{2m}}=\frac{2^{m}-1}{2^{m}}. \end{array}$$

*So, $h_{\mu l}\left(X\right)=1$, $h_{\mu l}\left(\Lambda ,\alpha \right)=1$ and $h_{\mu l}\left(\Lambda \right)=1$. Furthermore, if*

$$\begin{array}{c} X=(X_{1},X_{1},X_{1},\ldots ), \end{array}$$

*then $H_{\mu l}\left(X_{1},\ldots ,X_{1}\right)=H_{\mu l}\left(X_{1}\right)$ and $h_{\mu l}\left(X\right)=\frac{1}{2}$.*

**Figure 5:**
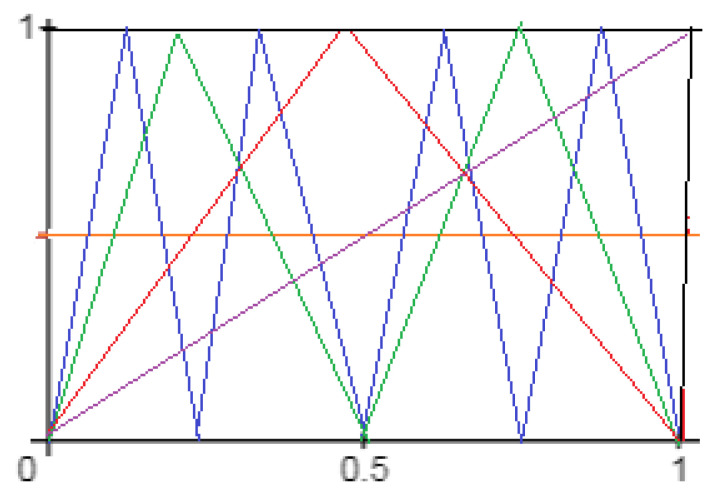
$x,\Lambda (x),\Lambda^{2}\left(x\right)\;\mathrm{and}\;\Lambda^{3}\left(x\right)$.

**Figure 6:**
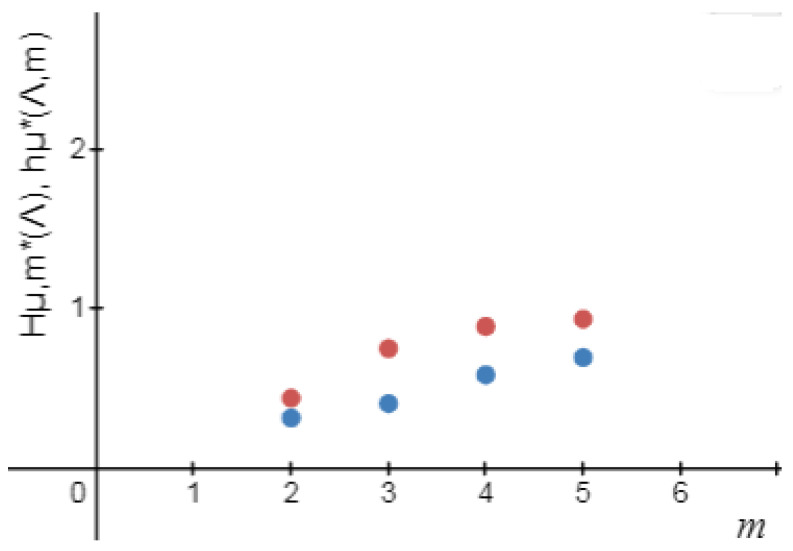
$H_{\mu l,m}^{*}\left(\Lambda \right),\;\mathrm{and}\;h_{\mu }^{*}\left(\Lambda ,m\right)$.

**Example** **3.** *Reference [1]. Consider the symmetric tent map in Example 2, we obtain (Figure 5 and Figure 6 and Table 3)*

$$\begin{array}{cc} \; & p_{\left(0,1\right)}=\left(0,\frac{2}{3}\right),\;p_{\left(1,0\right)}=\left(\frac{2}{3},1\right),\\ \; & p_{\left(0,1,2\right)}=\left(0,\frac{1}{3}\right),\;p_{\left(0,2,1\right)}=\left(\frac{1}{3},\frac{2}{5}\right),\;p_{\left(2,0,1\right)}=\left(\frac{2}{5},\frac{2}{3}\right),\\ \; & p_{\left(1,0,2\right)}=\left(\frac{2}{3},\frac{4}{5}\right),\;p_{\left(1,2,0\right)}=\left(\frac{4}{5},1\right),\;p_{\left(2,1,0\right)}=\emptyset ,\\ \; & p_{\left(0,1,2,3\right)}=\left(0,\frac{1}{6}\right),\;p_{\left(0,1,3,2\right)}=\left(\frac{1}{6},\frac{1}{5}\right),\;p_{\left(0,3,1,2\right)}=\left(\frac{1}{5},\frac{2}{9}\right)\cup \left(\frac{2}{7},\frac{1}{3}\right),\\ & p_{\left(3,0,1,2\right)}=\left(\frac{2}{9},\frac{2}{7}\right),\;p_{\left(0,2,1,3\right)}=\left(\frac{1}{3},\frac{2}{5}\right),\;p_{\left(2,0,3,1\right)}=\left(\frac{2}{5},\frac{4}{9}\right)\cup \left(\frac{4}{7},\frac{3}{5}\right),\\ \; & p_{\left(2,3,0,1\right)}=\left(\frac{4}{9},\frac{4}{7}\right),\;p_{\left(2,0,1,3\right)}=\left(\frac{3}{5},\frac{2}{3}\right),\;p_{\left(3,1,0,2\right)}=\left(\frac{2}{3},\frac{4}{5}\right),\\ \; & p_{\left(1,3,2,0\right)}=\left(\frac{4}{5},\frac{5}{6}\right),\;p_{\left(1,2,0,3\right)}=\left(\frac{6}{7},\frac{8}{9}\right),\;p_{\left(1,2,3,0\right)}=\left(\frac{5}{6},\frac{6}{7}\right)\cup \left(\frac{8}{9},1\right). \end{array}$$

*Therefore,*

$$\begin{array}{cc} \; & \sum_{\pi \in S_{2}}{\left(\mu \left(p_{\pi }\right)\right)}^{2}={\left(\frac{2}{3}\right)}^{2}+{\left(\frac{1}{3}\right)}^{2}=\frac{5}{9}≃0/556,\\ \; & H_{\mu l,2}^{★}\left(\Lambda \right)=1-\frac{5}{9}=\frac{4}{9}≃0/444,\\ \; & \sum_{\pi \in S_{3}}{\left(\mu \left(p_{\pi }\right)\right)}^{2}={\left(\frac{1}{3}\right)}^{2}+{\left(\frac{1}{15}\right)}^{2}+{\left(\frac{4}{15}\right)}^{2}+{\left(\frac{2}{15}\right)}^{2}+{\left(\frac{1}{5}\right)}^{2}=\frac{11}{45}≃0/244,\\ \; & H_{\mu l,3}^{★}\left(\Lambda \right)=1-\frac{11}{45}=\frac{34}{45}≃0/756,\\ \; & \sum_{\pi \in S_{4}}{\left(\mu \left(p_{\pi }\right)\right)}^{2}≃0.106,\\ \; & H_{\mu l,4}^{★}\left(\Lambda \right)≃0/894. \end{array}$$

*Furthermore,*

$$\begin{array}{c} h_{\mu l}^{★}\left(\Lambda \right)=\lim_{m\to \infty }H_{\mu l,m}^{★}\left(\Lambda \right)=1=h_{\mu l}\left(\Lambda \right). \end{array}$$

**Example** **4.** *Let I=[0,1] endowed with the measure ν,*

$$\begin{array}{c} \nu \left(A\right)=\chi_{A}\left(\frac{1}{2}\right)=\left\{\begin{array}{cc} 1\; & \;if\;\frac{1}{2}\in \;A\\ 0\; & \;if\;\frac{1}{2}\notin A, \end{array}\right. \end{array}$$

*and let f:[0,1]⟶[0,1] be a function. Then $h_{\nu l}\left(f,\alpha \right)=0$ for every partition α. Hence, $h_{\nu l}\left(f\right)=0$.*

## 5. Concluding Remarks

We introduced the concept of the logical entropy of random variables. In addition, we found a bound for the logical entropy of a random variable. We also extended the Shannon and permutation entropies to information sources. Finally, we used these results to estimate the logical entropy of the maps. In this article, we only introduced the concept of logical entropy for information systems. In future studies, researchers can find methods that calculate or estimate the numerical value of this type of entropy. It is pertinent to mention that, in the future, Rényi’s metric entropy and Rényi’s permutation entropy can be generalized for information sources. Another important problem is to extend this idea for quantum logical entropy, as it is a good direction to investigate the existence of such results.

## Figures and Tables

**Table 1:** Logical metric permutation entropy and metric permutation entropy [1] for the logistic map up to order m=3.

*m*	1	2	3
$H_{\mu l,m}^{★}\left(g\right)$	0	0/375	0/888
$h_{\mu }^{★}\left(g,m\right)$	0	0/28	0/39

**Table 2:** Logical metric entropy and metric entropy [1] for the tent map up to order m=3.

*m*	1	2	3	…	m
$H_{\mu l,m}\left(\Lambda \right)$	0	0/75	0.875	…	$1-\frac{1}{2^{m}}$
$h_{\mu }^{★}\left(\Lambda ,m\right)$	0	ln2	ln2	…	ln2

**Table 3:** Logical metric permutation entropy and metric permutation entropy [1] for the tent map up to order m=4.

*m*	1	2	3	4
$H_{\mu l,m}^{★}\left(\Lambda \right)$	0	0/444	0/756	0/894
$h_{\mu }^{★}\left(\Lambda ,m\right)$	0	0/32	0/41	0/59

## Appendix A

In this appendix, we prove the following results that we need in the paper.

**Lemma** **A1.**
*Let $M={\left[\theta_{ij}\right]}_{n\times n}$ be a matrix that $0\leq \theta_{ij}\leq 1$ for every 1≤i,j≤n and $\sum_{i,j}\theta_{ij}=1$, then*

$$\begin{array}{c} \sum_{i=1}^{n}{\left(\sum_{j=1}^{n}\theta_{ij}\right)}^{2}+\sum_{j=1}^{n}{\left(\sum_{i=1}^{n}\theta_{ij}\right)}^{2}\leq 1+\sum_{i,j}\theta_{ij}^{2}. \end{array}$$

**Proof.** Since

$$\begin{array}{cc} \sum_{i=1}^{n}{\left(\sum_{j=1}^{n}\theta_{ij}\right)}^{2}+\sum_{j=1}^{n}{\left(\sum_{i=1}^{n}\theta_{ij}\right)}^{2} & =\sum_{i,j}\theta_{ij}^{2}+{\left(\sum_{i,j}\theta_{ij}\right)}^{2}-2\sum_{i\neq k,j<l}\theta_{ij}\theta_{kl}\\ & =\sum_{i,j}\theta_{ij}^{2}+1-2\sum_{i\neq k,j<l}\theta_{ij}\theta_{kl}\leq 1+\sum_{i,j}\theta_{ij}^{2}, \end{array}$$

the assertion is proved. □

**Theorem** **A1.**
*Let $a_{ijk}$ be real numbers and $a_{ijk}\geq 0$ for every $1\leq i\leq n_{1}$, $1\leq i\leq n_{2}$ and $1\leq i\leq n_{3}$. Then*

(A1)
$$\begin{array}{c} 2\sum_{i,j}{\left(\sum_{k}a_{ijk}\right)}^{2}\leq \sum_{i,j,k}a_{ijk}^{2}+\sum_{i}{\left(\sum_{j,k}a_{ijk}\right)}^{2}. \end{array}$$

**Proof.** Since

$$\begin{array}{cc} 2\sum_{i,j}{\left(\sum_{k}a_{ijk}\right)}^{2} & =2\sum_{i,j}\left(\sum_{k,r}a_{ijk}a_{ijr}\right)=2\sum_{i,j,k,r}a_{ijk}a_{ijr}\\ \; & \leq \sum_{i,j,t,k,r}a_{ijk}a_{itr}=\sum_{i}{\left(\sum_{j,k}a_{ijk}\right)}^{2}\\ \; & \leq \sum_{i,j,k}a_{ijk}^{2}+\sum_{i}{\left(\sum_{j,k}a_{ijk}\right)}^{2}, \end{array}$$

which completes the proof of the theorem. □

**Lemma** **A2.**
*For an information source $X=(X_{1},X_{2},X_{3},\ldots )$,*

$$\begin{array}{c} \lim_{n\to \infty }H_{\mu l}\left(X_{n}|X_{n-1},\ldots ,X_{1}\right)=0. \end{array}$$

**Proof.** According to Theorem 9, the series $\sum_{n=1}^{\infty }H_{\mu l}\left(X_{n}|X_{n-1},\ldots ,X_{1}\right)$ converges and, thus, $\lim_{n\to \infty }H_{\mu l}\left(X_{n}|X_{n-1},\ldots ,X_{1}\right)=0.$ □
